# The rate, causes and predictors of ambulance call outs to residential aged care in the Australian Capital Territory: A retrospective observational cohort study

**DOI:** 10.1371/journal.pone.0311019

**Published:** 2024-09-30

**Authors:** Louise S. Cox, Mark Naunton, Gregory M. Peterson, Nasser Bagheri, Jake Paul Bennetts, Jane Koerner, Rachel Davey, Sam Kosari

**Affiliations:** 1 Discipline of Pharmacy, Faculty of Health, University of Canberra, Bruce, Canberra, ACT, Australia; 2 School of Pharmacy and Pharmacology, College of Health and Medicine, University of Tasmania, Hobart, TAS, Australia; 3 Health Research Institute, Faculty of Health, University of Canberra, Bruce, Canberra, ACT, Australia; University of Hong Kong, HONG KONG

## Abstract

Older people in residential aged care are susceptible to acute illness or injury which may necessitate an ambulance call out, assessment/treatment by a paramedic and transfer to a hospital emergency department. Understanding the case mix of residential aged care ambulance attendances is important for prevention strategies and for planning services. A retrospective observational closed cohort study was designed to investigate the characteristics of emergency ambulance call outs to 15 residential aged care sites in the Australian Capital Territory over a 12-month period. Data were collected from the local ambulance service and the aged care sites. Case load data were analysed to determine rates, clinical characteristics, ambulance attendance outcomes and the temporal distribution of call outs. A Poisson regression model was developed to investigate demographic, morbidity and medication-related risk factors associated with the number of ambulance call outs per resident. Annual ambulance call out costs were estimated. There were 1,275 residents, with 396 (31.1%) requiring at least one ambulance call out over 12 months. Of 669 ambulance attendances, the majority (87.0%) were transported to emergency departments. Trauma (23.9%), pain (16.9%) and infections (9.4%) were the most common primary assessments by the ambulance attendees. Cases/day were similar throughout the year and on weekdays compared to weekends/public holidays. The main predictors of ambulance call out were multi-morbidity, taking regular anticholinergic medicines, being male and younger age. Estimated costs of ambulance call outs/year were $475/resident and $40,375/residential aged care site. The most frequent primary assessments (trauma, pain, infections) may constitute priorities for developing prevention strategies and for treatment initiatives within residential aged care. Strategies to reduce anticholinergic medication prescribing may also be a potential intervention to decrease ambulance call outs and hospital emergency department demand. The ambulance usage data from this study may be useful to compare with future datasets to measure the impact of the introduction of new services.

## 1. Introduction

People aged over 65 years are a growing proportion of the population in Australia [1]. As people age, they may struggle to live independently in the community and so may require admission to residential aged care (RAC). In the year to June 2021, the number of permanent residents in RAC increased by 11% (from approximately 165,000 to approximately 184,000) [2] and this number is expected to continue to rise as the population ages.

Older people are living longer, are becoming frailer and have more co-morbidities [3]; they are therefore increasingly susceptible to presenting with acute clinical symptoms or injuries in RAC. Staff in RAC may determine that this requires an ambulance call out and subsequent clinical assessment by paramedics. The outcome of this primary clinical assessment may include no action, referral to a general practitioner (GP), onsite treatment or transfer to a hospital emergency department (ED) [4]. Transfer to ED by ambulance can be distressing for the resident and their family as it may be uncomfortable and disorientating [5]. Once in the ED, residents may have long waiting times, become anxious and be exposed to multiple staff, tests and examinations [5]. Not all ambulance call outs are appropriate. A systematic review of RAC transfers to ED found that between 4 to 55% were avoidable as clinical care should have been provided by the RAC [6]. Any subsequent admissions to hospital are also expensive and are associated with potential health complications for older people [5]. Additionally, unecessary ambulance call outs and ED transfers put pressure on the already stretched ambulance and hospital systems, and therefore need to be avoided whenever possible.

Ambulance service data about clinical assessments, onsite treatment and ED transfers can assist RAC providers to improve the quality of care delivered. Onsite treatment by ambulance paramedics can be indicated for myocardial infarction, cerebral vascular accident, spinal injuries, drug overdose, mental illness, fractures, head injuries, abdominal bleeding, internal or external trauma injuries [7]. Potentially avoidable ambulance call outs and ED transfers from RAC may indicate potential deficiencies in RAC protocols or gaps in service delivery. These include insufficient access to primary healthcare staff [6, 8], RAC staff shortages [6, 8, 9], staff training issues [6, 9], poor or non-adherence to advance care planning [6, 8], poor end of life care [6, 8, 9] and suboptimal clinical care [9].

Data about presentations to ED of residents from RAC are important for understanding the healthcare utilisation of residents and for monitoring the quality of the sector. Such data was lacking in Australia until it was examined by the Royal Commission into Aged Care Quality and Safety [10]. Data were obtained from hospital and ED in every State and Territory in Australia. The Commission’s research paper determined that 36.9% aged care residents presented to ED and 37% residents were admitted to hospital (public and private) at least once during 2018/2019 [10]. The most common reasons for hospital admissions and ED presentations were respiratory disease, injuries, circulatory disease, dialysis and ‘symptoms and signs’ [10]. Whilst these data provided a valuable insight into the prevalence and conditions that necessitated a hospital transfer, they did not give information about the frequency of ambulance call outs, whether weekends or public holidays generate more ambulance call outs, those acute presentations that could be treated by paramedics or staff within the RAC, and incidents where a decision is made not to transfer the resident to ED.

The role of medicines in potentially avoidable ambulance call outs and ED transfers is also worthy of consideration. Residents of RAC are generally frail older people with complex co-morbidities who take multiple medications. Studies report that residents take on average between 7 and 11 medicines each day [11], putting them at high risk of adverse drug events which may result in transfer to ED by ambulance. The quality of prescribing is therefore important. Some medications are classified as potentially inappropriate medications (PIMs), for which the risk of adverse events outweighs the clinical benefits, especially in the elderly [12]. International studies have determined that the use of PIMs in older people is associated with an increased risk of hospitalisation [13]. This is especially problematic in RAC, where a systematic review identified that 43.2% of medications used have been classified as PIMs [14]. Antipsychotics and drugs with anticholinergic properties are PIMs that are particularly associated with risk of harm in the elderly [15], so the prescribing of these agents could be expected to be associated with ambulance call outs.

The objective of this study was to determine the rates and most common causes of ambulance call outs from a sample of RAC sites in the Australian Capital Territory (ACT). Secondary objectives were to: determine whether demographic-, morbidity- or medication-related factors were associated with higher numbers of call outs; determine whether there was a difference in call out rates between the seasons, weekends/public holidays and weekdays; identify the paramedic assessments that were treated in RAC or required ED transfer; and calculate the cost of ambulance call outs to RAC.

## 2. Materials and methods

This study is reported in accordance with the Strengthening the Report of Observational Studies in Epidemiology (STROBE) Statement [16]. (S1 Checklist in Supporting Information)

### 2.1 Design

This retrospective observational study was an analysis of ambulance data obtained during the Pharmacists in Residential Aged Care Facilities (PiRACF) study, which evaluated the effects of integrating on-site pharmacists into RAC to improve medication management [17, 18].

### 2.2 Study sample

The study was conducted in the ACT, which had an estimated population of 454,499 in 2021, with 62,203 (13.7%) aged over 65 years [19]. Fifteen RAC sites (out of 25 RACs in ACT) were recruited to PiRACF for a duration of 12 months in a staggered manner [18]. Both control and intervention RAC sites from the 15 PiRACF sites were included in this pooled analysis. All permanent residents of the PiRACF RAC sites were included unless they specifically requested their data not to be used. Residents who moved to RAC during the study period were excluded.

The characteristics of ambulance personnel attending the RAC sites varied from case to case. Intensive care paramedics, ambulance paramedics and extended care paramedics are employed by ACT Ambulance Service. In most cases a double crew ambulance attended the RAC site, noting that ACT does also operate single response units. If an extended care paramedic attended, then it was a solo paramedic attendance. All the RAC sites had access to local initiatives that may have reduced ambulance call outs. These included nurse practitioners, a palliative care team of advanced practice registered nurses/nurse practitioners, and variable access to a Geriatric Rapid Acute Care Evaluation (GRACE) team. GRACE comprises a team of clinicians who assess and develop a care plan for RAC residents, with an aim to prevent hospital admissions [20]. Additionally, residents may have had specific clinical indications where treatment was not appropriate according to their advance care directives.

### 2.3 Data collection

Data were obtained on 12^th^ January 2022 from the ACT Ambulance Service on callouts and transport of residents to ED from each of the 15 study RAC site for 12 months (over the period each RAC site participated in the PiRACF study) [18]. Demographic and medication data were obtained from the 15 RAC sites. The data were de-identified prior to analysis.

### 2.4 Analysis

Analysis of the data was conducted using password-protected Microsoft Excel workbooks by one researcher (JB) and this was checked by a second researcher (LC). For each episode, the following data were extracted: date; age; gender; the final primary assessment of the acute health problem by the emergency respondents; whether the resident was treated by emergency respondents; if the resident was subsequently transferred by ambulance to the ED; Charlson Comorbidity Index (CCI) [21]; whether a PIM [12] was prescribed as part of the regular drug regimen (binary); Anticholinergic Cognitive Burden (ACB) score of medications [15]; and whether they were prescribed any antipsychotic medications (binary). The American Geriatrics Society has defined a PIM in their Beers® criteria as a medication where the risk of adverse effects is greater than the expected clinical benefits for an elderly individual [12]. The Beers® criteria were modified to incorporate medications licensed in Australia [22]. An experienced clinical pharmacist with medication review skills applied the Beers® Criteria to residents’ medications to identify PIMs [23]. Training and ongoing review was provided by two researchers (MN, SK) who are pharmacists renowned for their expertise in identifying PIMs [23]. The ACB can be used to define the degree of negative cognitive effects expected from medication prescribed for an individual [15]. Annual ambulance call out costs were estimated per resident and per RAC site using the data provided by ACT Emergency Services Agency [24].

Descriptive analyses comprised producing raw counts and percentages for age groupings, gender and final primary assessment. Treatment by emergency respondents and transport to hospital were binary (yes/no) variables. For temporal characteristics the data were separated into seasons, weekdays and weekends/public holidays for comparison of workload patterns. Seasons were defined as spring (September-November), summer (December-February), autumn (March-May) and winter (June-August).

A univariate analysis was conducted to determine whether there was significant associations between the number of ambulance calls and residents’ age, male gender, prescribed antipsychotic medication, CCI, prescribed PIM and ACB score of medications. A Poisson regression model was developed to investigate the demographic, morbidity and medication-related factors associated with the number of ambulance call outs per resident. The model estimated incident rate ratios (IRRs), robust standard error, p values and 95% confidence intervals (95% CIs). Stata (release 16.1; Stata Statistical Software, College Station, TX: StataCorp LLC. USA) was used for the statistical analysis.

### 2.5 Ethical approval

This study was part of the PiRACF study and ethics approval was obtained from the University of Canberra Human Research Ethics Committee (UC HREC Reference: 2007), the ACT Health Human Research Ethics Committee (ACT Health HREC Reference: 2019/ETH13453) and Calvary Public Hospital Bruce Human Research Ethics Committee (Calvary HREC Reference: 30–2019). Written consent to participate was gained at the facility level, rather than the resident level. Residents were able to opt out of having their data included in the study, and the process on how to do this was provided to residents and families. This consent process follows Australian National Health and Medical Research Council guidelines [25] and is consistent with comparable studies conducted in Australia [26, 27].

## 3. Results

**Fig 1:**
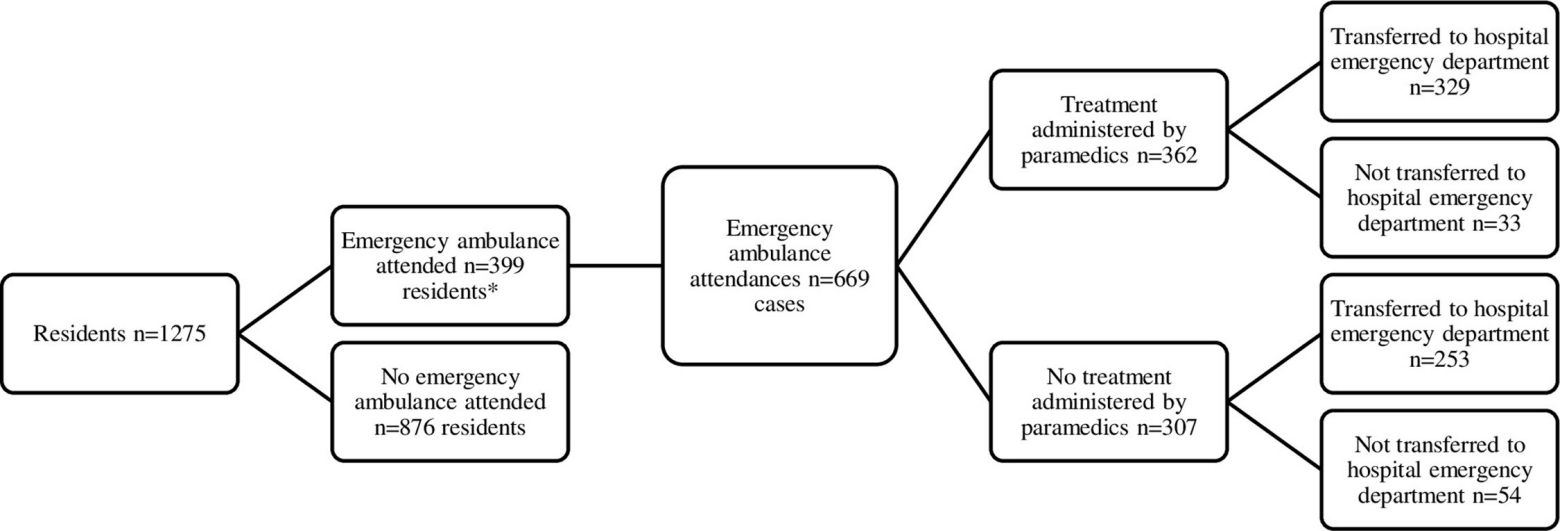
Flow diagram describing participation in the study. * Residents could have more than one ambulance attendance.

Data was collected between 24^th^ January 2020 and 11^th^ November 2021. A total of 1,275 residents were enrolled in the study, with 396 (31.1%) of these residents requiring at least one ambulance call out during 12 months (Table 1). There were 669 ambulance call outs to the 15 RAC sites (Fig 1). The majority of residents (66.6%) that required an ambulance call out did so only once during the study period, but some residents received multiple ambulance attendances, with one resident needing 15 ambulance call outs (Table 2). Just over half (54.1%) of call outs were treated by the attending ambulance crew at the RAC site. Many cases (87.0%) were transported to the ED by ambulance (Table 3); this was slightly lower for trauma and cardiovascular issues than for pain and infections. The rate of cases per day, percentage treated in RAC and percentage transferred to ED were similar on usual working days compared to weekends and public holidays. Trauma (23.9%), pain (16.9%) and infections (9.4%) were the most common primary assessments by the ambulance attendees (Table 1; see S1 Table in Supporting Information for a comprehensive breakdown). The cost of ambulance call outs per year was estimated to be $40,375 per RAC site and $475 per resident (Table 4).

**Table 1:** Demographics of participants from the 15 residential aged care sites.

	Total, n (%)		Total, n (%)
**All residents**	1275 (100%)	**Antipsychotic medications prescribed**	
**Age (years)**		Yes	214 (16.8%)
Less than 65	1 (0.1%)	No	1061 (83.2%)
65 to 69	25 (2.0%)	**Charlson comorbidity index (CCI)**	
70 to 74	93 (7.3%)	0	145 (11.4%)
75 to 79	117(9.2%)	1	346 (27.1%)
80 to 84	213 (16.7%)	2	272 (21.3%)
85 to 89	284 (22.3%)	3+	512 (40.2%)
90 to 94	299 (23.5%)	**Potentially Inappropriate Medications (PIM)**	
95 to 99	204 (16.0%)	Yes	871 (68.3%)
100 to 104	34 (2.7%)	No	404 (31.7%)
105 to 109	5 (0.4%)	**Anticholinergic Cognitive Burden (ACB) score**	
**Gender**		0	639 (50.1%)
Male	436 (34.2%)	1–2	385 (30.2%)
Female	839 (65.8%)	3+	251 (19.7%)

**Table 2:** Demographics of residents related and number of call outs.

Number of calls	Number of residents (%)	Age in years median (range)	Number of females (%)
	n = 1275	88 (64–108)	n = 839 (65.8%)
0	876 (68.7%)	88 (64–108)	602 (68.7%)
1	256 (20.1%)	88 (67–105)	162 (63.3%)
2	92 (7.2%)	86.5 (68–105)	52 (56.5%)
3	21 (1.6%)	83 (71–94)	11 (52.4%)
4	14 (1.1%)	92 (69–101)	6 (42.9%)
5	5 (0.4%)	82 (76–90)	3 (60.0%)
6	5 (0.4%)	82 (70–95)	1 (20.0%)
7	2 (0.2%)	84 (80–88)	0 (0.0%)
8	1 (0.1%)	73 (73)	0 (0.0%)
9	2 (0.2%)	85 (84–86)	1 (50.0%)
15	1 (0.1%)	81 (81)	1 (100%)

**Table 3:** Characteristics of ambulance call outs to the 15 residential aged care sites.

Final Primary assessment	Total (%)	Treated at RAC (%)#	Transported to ED# #(%)
Trauma	160 (23.9%)	86 (55.6%)	130 (81.3%)
Pain	107 (16.0%)	71 (66.4%)	99 (92.5%)
Infections	63 (9.4%)	44 (69.8%)	62 (98.4%)
Respiratory	37 (5.5%)	30 (81.1%)	35 (94.6%)
Gastrointestinal	34 (5.1%)	20 (58.8%)	33 (97.1%)
Genitourinary	33 (4.9%)	11 (33.3%)	29 (87.9%)
Neurological	28 (4.2%)	12 (42.9%)	27 (96.4%)
Cardiovascular	27 (4.0%)	16 (59.2%)	22 (81.5%)
Altered mental state	26 (3.9%)	17 (65.4%)	22 (84.6%)
Other	154 (23.0%)	55 (35.7%)	123 (79.9%)
**Ambulance call outs**	**N (rate/day)**	**Treated at RAC (%)**	**Transported to ED (%)** ^**#**^
Total (658 days)	669 (1.0)	362 (54.1%)	582 (87.0%)
Working day (451 days)	464 (1.0)	246 (53.0%)	409 (90.5%)
Weekend/public holidays (207 days)	205 (1.0)	116 (56.6%)	173 (84.4%)
Summer (127 days)	177 (1.4)	90 (50.8%)	149 (84.2%)
Autumn (184 days)	159 (0.9)	92 (57.9%)	141 (88.7%)
Winter (184 days)	158 (0.9)	84 (53.2%)	139 (88.0%)
Spring (163 days)	175 (1.1)	96 (58.5%)	153 (87.4%)

# Note residents can be both treated at the aged care home and then transported to the emergency department

Treatment at RAC can comprise that for myocardial infarction, cerebral vascular accident, spinal injuries, drug overdose, mental illness, fractures, head injuries, abdominal bleeding, internal or external trauma.

RAC residential aged care

ED emergency department

**Table 4:** Costs (AUD) of ambulance call outs to residential aged care.

Ambulance activity	Number of cases	Cost per case ($)	Cost ($)
Treatment and transport	329	1,000^a^	329,000
Treatment only (no transport)	33	693^a^	22,869
Transport only (no treatment)	253	1,000^a^	253,000
No treatment/transport	54	140^b^	7,560
**Total**	**669**		**605,625**
Mean cost per aged care site (n = 15)			40,375
Mean cost per resident (n = 1,275)			475

Notes:

^a^ ACT ambulance service rates in August 2021.

^b^ Although ambulance call outs which did not require treatment/transport are not charged, the travel time cost was estimated to be an average of $140 per call out ($14 per km and assuming a 10km ride)

Univariate analysis identified significant associations between the number of ambulance calls and residents’ age (a negative association; p = 0.001), male gender (p<0.001), CCI (p<0.001) and ACB score of medications (p = 0.001). The findings were further confirmed using Poisson regression analysis that showed the main predictors of ambulance calls were multi-morbidity measured by CCI, ACB score of regular medicines, being a male, and being younger (Table 5). For each one unit increase in the CCI and in the score of ACB for regular medicines, the incidence of ambulance calls is expected to increase by 12% and 11%, respectively, holding the other variables constant. The ambulance call out rate ratio for males was 1.43 times of that for females. Additionally, for each one-year increase in age the ambulance call rate ratio decreased by 0.02%.

**Table 5:** Univariate and Poisson regression analysis of factors associated with number of ambulance call outs (per resident).

	Univariate analysis					Poisson regression analysis				
Factors	IRR	Robust standard error	z	p value	(95% CI)	IRR	Robust standard error	z	p value	(95% CI)
Age	0.98	0.006	-3.21	0.001*	(0.96, 0.99)	0.98	0.006	-2.35	0.01*	(0.97, 0.99)
Male gender	1.55	0.18	2.95	0.000*	(1.23, 1.96)	1.43	0.17	2.95	0.00*	(1.12, 1.81)
Prescribed antipsychotic medication	1.09	0.14	0.70	0.483	(0.84, 1.42)	0.73	0.13	-1.66	0.09	(0.55, 1.11)
CCI	1.13	0.03	4.11	0.000*	(1.06, 1.20)	1.12	0.03	3.45	0.00*	(1.05, 1.19)
Prescribed potentially inappropriate medication (PIM)	1.19	0.15	1.40	0.160	(0.83, 1.39)	1.07	0.14	0.56	0.57	(0.83, 1.39)
Anticholinergic Cognitive Burden (ACB) score of medications	1.10	0.03	3.20	0.001*	(1.04, 1.18)	1.11	0.04	2.63	0.00*	(1.02, 1.19)

All estimates are adjusted for age and gender. Medications refer to medications taken regularly and do not include ‘PRN’ medications.

*- significant p values

IRR Incidence rate ratio

CI Confidence Interval

## 4. Discussion

This study analysed RAC cases referred to the ambulance service in order to understand the frequency, case-mix and workload for RAC, the ambulance service and the ED. Trauma, pain and infections were the most common primary assessments by the ambulance attendees. The main predictors of ambulance call out were multi-morbidity, taking regular anticholinergic medicines, being male and being younger. This is important as a knowledge of the frequency and case types can identify gaps in care, inform training of staff and can stimulate initiatives to treat residents within RAC. An understanding of the workload data can assist with resource planning for staff levels and skill mix within the ambulance service, RAC and ED.

Ambulance call outs to RAC were relatively frequent, with an average of one per day across the RAC sites and involving almost one-third of residents during the study period. The mean cost of ambulance call outs was estimated to be $475 per resident per year. The decision to call an ambulance by the care team is complex and may be influenced by the perception that hospitalisation may improve the outcome for the resident, or factors unrelated to outcomes such as the ability or confidence of RAC staff to treat a condition, inadequate advance care planning, the expectations of family or by litigation fears of RAC leadership [28]. In addition, it is possible that RAC facilities which are inadequately resourced may have fewer skilled staff and therefore make decisions which impact the quality of care of their residents [29], which may include calling an ambulance with subsequent transfer to hospital [28]. Understanding the reasons for calling the ambulance may identify quality issues within RAC and this could be explored in future studies.

Reducing the frequency of ambulance call outs to RAC is desirable. Novel services that may reduce ambulance call outs include after-hours telehealth provided by ED physicians [30], increasing RAC staff competency in delivering acute care [31], enhanced co-ordination of care [32], employing GPs in RAC [33], and nurse-practitioner led care [34]. A recent Cochrane review concluded that different care models may have little or no effect on ED visits compared to usual care but may decrease unplanned hospital admissions [35], although research is lacking on the effects of novel services on ambulance usage patterns.

Clinical presentations to the ambulance attendees varied, with trauma, pain and infections being the most common. These findings are consistent with the emergency ambulance demands from RAC reported in other studies in Australia [36, 37]. Whilst we did not have access to clinical assessment details, it is reasonable to assume that trauma was often associated with falls. Strategies to reduce falls [38, 39] within RAC are therefore important and there is evidence that pharmacist-conducted medication reviews in RAC are one approach that may reduce falls [40]. There is also the possibility that in some cases falls, pain and infections may be treated outside of hospital if access to primary health care teams are enhanced or alternative acute care pathways are introduced [28]. The most common identified infections were chest infections, urinary tract infections and pneumonia. Treatment of pneumonia within RAC using a clinical pathway (chest X-ray, oxygen saturation monitors, oral antibiotics, rehydration, and monitoring by a nurse) was demonstrated to reduce hospitalisations compared to usual care in a study in Canada [41] and therefore may also reduce ambulance usage. Antibiotic prescribing for urinary tract infections in RAC can be improved by decision tools integrated within electronic health records, together with support from physicians and nurses [42], although there was not a statistically significant reduction in hospitalisations in the small study. Another approach that may improve the treatment of infections in RAC is antimicrobial stewardship, which is a role that can be conducted by pharmacists [43]. As infections are a significant contribution to ambulance call outs in this population it is worthwhile to investigate services to address this issue.

A transfer to ED resulted from the majority of ambulance attendances, but the transfer rate of residents to ED was only marginally lower (87% v 91%) than a comparable study in Melbourne [36]. The rate of cases per day, percentage treated in RAC and percentage transferred to ED were similar on usual working days compared to weekends and public holidays, and this is consistent with other studies [36, 37].

Increased usage of ambulance call outs were associated with younger age, morbidity measured by CCI, the ACB score of regular medicines and being male. The age, morbidity and gender differences in ambulance call outs may be explained by the uptake of advance care directives (preferences for medical treatment including circumstances where treatment is to be refused) which have been found to be higher for those of older age and females [44]. Additionally patients with higher comorbidity, older age and a higher ACB score may experience higher mortality rates so make fewer future ambulance calls outs. Greater ambulance use by males was also found in the analysis of 2009–2013 data from Victoria, with possible explanations cited including that genders have differing clinical signs and that males have a lower threshold to seek assistance compared to females [45]. High rates of comorbidity and polypharmacy were also prevalent in residents with ambulance call outs in Victoria [37]. This is unsurprising as polypharmacy is linked to morbidity and disease severity [46]. In the Victorian study an average of 7.9 medications were prescribed, many contributing to ACB, with 25% taking opioids, 24% sedatives (mainly benzodiazepines), 20% antidepressants and 14% antipsychotics, but the appropriateness of medication was not determined in that study [37]. Polypharmacy and PIM prescribing have been associated with increased hospitalisation from RAC [46], but our study did not link PIM use to increased ambulance call outs. The reason that increased PIMs were not linked to ambulance call outs may be due to the type of PIM prescribed in our study population compared to other studies. The adverse effects of anticholinergics on the elderly are well recognised (e.g. confusion, falls and urinary retention) [15, 47], so the associated increase in ambulance call outs found in our sample is to be expected.

Utilising pharmacists, the medication experts, in RAC may be worthwhile to address the identified medication-related links to ambulance call outs. Currently, pharmacists in USA, England and Australia predominantly conduct medication reviews in aged care homes but also are involved in other activities, including staff education, implementing policies and procedures, collaboration with GPs and community pharmacists, antimicrobial stewardship and conducting audits [43]. There is limited data on the effectiveness of pharmacist services in this setting. A randomised control trial of pharmacist independent prescribers in care homes in the United Kingdom, determined that deprescribing of anticholinergic and sedative drugs occurred over a six-month follow-up period, but there was no change in clinical outcome measures that included falls, hospitalisation, health service use and mortality [48].

### 4.1. Strengths and limitations

This study is subject to some limitations. The study participants were from 15 RAC sites in one largely urban Australian territory with good access to public hospitals; thus, the findings may not be generisable to regional, rural and remote areas. The primary assessment category of ‘other’ was recorded by the ambulance paramedics for almost a quarter of cases. More extensive data, including who contacted the ambulance service, the time of the call, the appropriateness of the call, the accuracy of the final primary assessment by the paramedics, subsequent hospital admissions, the appropriateness of any ED transfer and whether residents returned to the RACs may have enhanced the study. However, the use of ambulance data provides a broader insight than that provided by EDs as it includes the acute presentations treated within the RAC but not transferred to hospital.

## 5. Conclusion

This study investigated the rates, clinical characteristics and risk factors for ambulance call outs to RAC. The data provides a useful baseline for resource allocation and for measuring changes in quality of care in RAC. Future research could include the effect of services to reduce the identified modifiable risk factors or could compare the clinical outcomes, acceptability and resource implications of ambulance transport to an ED versus telephone triage or paramedic assessment with referral to alternate health services.

## Supporting information

S1 Checklist STROBE statement—checklist of items that should be included in reports of cohort studies. (DOC)

S1 Table Comprehensive breakdown of ambulance call outs. (DOCX)
