# Supplementary material for: The rate, causes and predictors of ambulance call outs to residential aged care in the Australian Capital Territory: A retrospective observational cohort study
Source: PLoS One. 2024 Sep 30;19(9):e0311019. doi: 10.1371/journal.pone.0311019 (PMC11441681; doi:10.1371/journal.pone.0311019)
Supplement: S1 Table — (DOCX) [file pone.0311019.s002.docx]

**Supporting Information**

**S2 Table: Comprehensive breakdown of ambulance call outs**

|  |  |  |  |
| --- | --- | --- | --- |
| **Final primary assessment** | **Total (% total FPA)** | **Treated at RAC (% FPA category)** | **Transported to ED (% FPA category)** |
| **Trauma** | **160 (23.9%)** | **86 (55.6%)** | **130 (81.3%)** |
| Laceration | 58 | 34 | 44 |
| Fracture/s | 27 | 23 | 27 |
| Bruising / Haematoma | 22 | 6 | 15 |
| Head Injury | 14 | 9 | 14 |
| Abrasion / Graze | 13 | 3 | 7 |
| Epistaxis | 8 | 4 | 7 |
| Open Wound | 8 | 3 | 7 |
| Soft Tissue Injury | 5 | 1 | 4 |
| Dislocation | 3 | 2 | 3 |
| Eye Injury / Problem | 1 | 0 | 1 |
| Face Injury / Problem | 1 | 1 | 1 |
| **Pain** | **107 (16.0%)** | **71 (66.4%)** | **99 (92.5%)** |
| **Infection** | **63 (9.4%)** | **44 (69.9%)** | **62 (98.4%)** |
| Febrile | 17 | 12 | 17 |
| Chest Infection | 13 | 10 | 13 |
| Sepsis | 11 | 11 | 11 |
| Infection - Other / Not Listed | 10 | 3 | 10 |
| Pneumonia | 6 | 6 | 6 |
| Cellulitis | 4 | 2 | 4 |
| Wound Inflammation / Infection | 1 | 0 | 1 |
| Respiratory Tract Infection | 1 | 0 | 0 |
| **Cardiovascular** | **27 (4.0%)** | **16 (59.2%)** | **22 (81.5%)** |
| Hypotension | 9 | 6 | 9 |
| Hypertension | 6 | 1 | 5 |
| Acute Coronary Syndrome | 5 | 4 | 5 |
| Cardiac Arrest | 3 | 3 | 1 |
| Arrhythmia | 2 | 0 | 0 |
| Cardiac Failure | 1 | 1 | 1 |
| Acute Myocardial Infarction | 1 | 1 | 1 |
| **Respiratory** | **37 (5.5%)** | **30 (81.1%)** | **35 (94.6%)** |
| Short of Breath | 27 | 23 | 27 |
| Chronic Obstructive Pulmonary Disease | 3 | 3 | 3 |
| Cough | 3 | 1 | 2 |
| Pulmonary Aspiration | 2 | 2 | 2 |
| Acute Pulmonary Oedema | 1 | 0 | 1 |
| Asthma | 1 | 1 | 0 |
| **Gastrointestinal** | **34 (5.1%)** | **20 (58.8%)** | **33 (97.1%)** |
| Vomiting | 13 | 9 | 13 |
| Nausea | 7 | 4 | 6 |
| Bowel Obstruction | 4 | 2 | 4 |
| Abdominal Distension | 3 | 2 | 3 |
| Gastrointestinal Problem | 3 | 2 | 3 |
| Haematemesis | 1 | 1 | 1 |
| Constipation | 1 | 0 | 1 |
| Diarrhoea | 1 | 0 | 1 |
| Melaena | 1 | 0 | 1 |
| **Altered mental state** | **26 (3.9%)** | **17 (65.4%)** | **22 (84.6%)** |
| Altered Conscious State | 12 | 9 | 11 |
| Confusion | 9 | 5 | 9 |
| Alcohol Intoxication | 2 | 1 | 0 |
| Psychiatric Episode | 2 | 2 | 2 |
| Emotional Distress | 1 | 0 | 0 |
| **Genitourinary** | **33 (4.9%)** | **11 (33.3%)** | **29 (87.9%)** |
| Urinary Catheter Problem | 18 | 4 | 14 |
| Urinary Tract Infection | 7 | 4 | 7 |
| Urine Retention | 2 | 1 | 2 |
| Haematuria | 2 | 1 | 2 |
| Dysuria | 1 | 0 | 1 |
| Incontinence - Urinary | 1 | 0 | 1 |
| Prolapse | 1 | 1 | 1 |
| PV Bleeding | 1 | 0 | 1 |
| **Neurological** | **28 (4.2%)** | **12 (42.9%)** | **27 (96.4%)** |
| Stroke | 10 | 3 | 10 |
| Seizure/s / Convulsion/s | 10 | 7 | 10 |
| Faint | 3 | 1 | 2 |
| Weakness | 2 | 0 | 2 |
| Headache | 1 | 0 | 1 |
| Post Ictal | 1 | 1 | 1 |
| Visual Disturbance / Loss | 1 | 0 | 1 |
| **Other** | **154 (23.0%)** | **55 (35.7%)** | **123 (79.9%)** |
| Other - Specify | 45 | 17 | 44 |
| No Problem Identified | 31 | 8 | 15 |
| Unknown Problem | 29 | 10 | 26 |
| Blank | 14 | 0 | 14 |
| Mobility Problem | 9 | 3 | 7 |
| Swollen Limb | 8 | 5 | 6 |
| Deceased | 5 | 4 | 0 |
| Hyperglycaemia | 3 | 1 | 3 |
| Hypoglycaemia | 3 | 2 | 2 |
| Social Problem | 2 | 1 | 1 |
| Dehydration | 2 | 2 | 2 |
| Overdose | 1 | 1 | 1 |
| Renal Failure | 1 | 0 | 1 |
| Allergic Reaction | 1 | 1 | 1 |
